# Comorbidities and the Risk of Late-Stage Prostate Cancer

**DOI:** 10.1100/tsw.2006.383

**Published:** 2006-07-28

**Authors:** Steven T. Fleming, Kathleen McDavid, Kevin Pearce, Dmitri Pavlov

**Affiliations:** ^1^ University of Kentucky College of Public Health, Lexington, KY, USA; ^2^ Centers for Disease Control and Prevention, Atlanta, GA, USA; ^3^ University of Kentucky, Family Practice & Community Medicine, Lexington, KY, USA; ^4^ Pfizer Inc., New London, CT, USA

**Keywords:** comorbidity, race, claims data, Medicare, prostate cancer, stage of illness

## Abstract

The degree to which comorbidities affect the diagnosis of prostate cancer is not clear. The purpose of this study was to determine how comorbidities affect the stage at which prostate cancer is diagnosed in elderly white and black men. We obtained data from the Surveillance, Epidemiology, and End Results program of the National Cancer Institute merged with Medicare claims data. For each patient, we estimated associations between stage of disease at diagnosis and each of the 27 comorbidities. The sample included 2,489 black and 2,587 white men with staged prostate cancer. Coronary artery disease, benign hypertension, and dyslipidemia reduced the odds of late-stage prostate cancer. A prior diagnosis of peripheral vascular disease, severe renal disease, or substance abuse increased the odds of being diagnosed with late-stage disease. The study shows some effect modification by race, particularly among white men with substance abuse, cardiac conduction disorders, and other neurologic conditions. The strongest predictors of late-stage prostate cancer diagnosis for both white and black men were age at diagnosis of at least 80 years and lack of PSA screening. Comorbidities do affect stage at diagnosis, although in different ways. Four hypotheses are discussed to explain these findings.

## INTRODUCTION

Prostate cancer represents a significant disease burden for elderly men, especially those who are black[1,2,3]. Most prostate cases (80%) are diagnosed in men who are at least 65 years of age, and whose disease is at regional stage and poor histologic grade[3]. Black men have a 60% higher incidence rate of prostate cancer than white men[3]. Over a 5-year period starting in the late 1980s, the incidence rate of prostate cancer increased over 100% for both white and black men[3]. The widespread use of the prostate-specific antigen (PSA) blood test, introduced in 1986, probably contributed greatly to this steep increase in the incidence of prostate cancer, especially locally staged cancer, in the U.S. during this time period[3,4,5], although no causal link between screening and either incidence or mortality has been established[6].

While 75% of prostate cancer patients will not die of the disease, many will experience significant morbidity from urinary, bowel, and sexual dysfunction[7,8,9,10,11,12,13,14]. The financial costs associated with prostate cancer treatment are also substantial[13,15,16,17,18]. Comorbidities, or coexisting illnesses, are prevalent among the elderly. While some evidence suggests that comorbid illness may influence both choice of treatment[19,20,21,22] and survival[21,23,24,25], the degree to which these illnesses affect the screening and recognition of prostate cancer is unclear. Comorbidities may compete or distract physician attention from cancer screening, or increase the likelihood of being screened due to more contact with health care providers[26,27,28,29]. The purpose of this study was to determine which comorbidities affect the stage at which prostate cancer is diagnosed and whether these effects differ in white and black men.

## METHODS

We obtained Internal Review Board (IRB) approval from the University of Kentucky to obtain data from the Surveillance, Epidemiology and End Results (SEER) program of the National Cancer Institute (NCI), which collects and publishes cancer incidence and survival data from 11 population-based cancer registries and 3 supplemental registries covering approximately 14% of the U.S. population. The database includes information on more than 2.5 million *in situ* and invasive cancer cases. The data for this study were drawn from the SEER clinical and demographic data on cancer cases in 5 states (Connecticut, Hawaii, Iowa, New Mexico, and Utah) and 6 metropolitan areas (Los Angeles, San Francisco-Oakland, San Jose, Detroit, Seattle, and Atlanta). We used SEER data linked to Medicare claims, jointly administered by NCI and the Centers for Medicare and Medicaid Services[30].

We identified all (n = 7,209) black men diagnosed with prostate cancer in 1993–1995. Subjects were then excluded for a number of reasons: age less than 67 years at diagnosis (31.2%), health maintenance organization (HMO) membership (18.8%), incomplete Medicare parts A and B coverage (7.6%), no month of cancer diagnosis, diagnosis from autopsy or death certificate, and prostate cancer diagnosis earlier than 1993 (all less than 1.0% each). Some patients were excluded for more than one reason. Of the remaining 2,931 black men with at least 2 years of Medicare claims data prior to cancer diagnosis, 441 (15%) had no definitive stage recorded, leaving a final sample of 2,490 staged black men, 2,489 of whom had complete covariate data (i.e., sociodemographic variables).

For the purpose of interracial comparisons, we also identified all white men diagnosed with prostate cancer (n = 48,253), and excluded those who were less than 67 years of age at diagnosis (23.5%), belonged to an HMO (17.7%), had incomplete Medicare A and B coverage (4.8%), had no month of cancer diagnosis, or whose prostate cancer diagnosis was earlier than 1993. From the remaining 25,596 white men, we randomly chose a sample of 3,000 and then eliminated 51 who were diagnosed by autopsy or death certificate, and 362 (12.3%) with no definitive stage recorded, yielding a final sample of 2,587 white men with staged prostate cancer.

We identified which, if any, of 27 comorbidities[31] were present in each member of the sample based on at least one diagnosis within 2 years prior to the prostate cancer diagnosis. We obtained data on comorbidities from inpatient, outpatient, and physician-supplier claims. For the physician data, we included only claims in which the HCFA-type service code was identified as “medical,” “surgical,” or “consultation” because such diagnoses were more likely to be recorded accurately and “physician-directed” than diagnoses coded as “diagnostic radiology” or “diagnostic laboratory”, for example.

We made a couple of modifications to SEER staging. For this analysis, we considered men with cancers staged *in situ* or local as having early-stage disease and those with regional spread or distant cancer as having late-stage disease. However, staging by SEER was not consistent for some years of our study, as a significant percentage of cases (21% of black men) received a code of local/regional, rather than one or the other. Instead of eliminating these cases, we restaged the disease based on an algorithm involving the degree to which the tumor had spread beyond the primary site. Extension of the tumor is a two-digit code that characterizes the degree to which the tumor has spread beyond the primary site. The *in situ* stage must have a “00” extension and a “negative” or “not stated” lymph node status. Local disease must have an extension within the range 01–39 and “negative” or “not stated” lymph node status. We restaged to regional those patients with a 01–39 extent of disease and “positive regional nodes” or “positive nodes NOS”, or a 40–79 extent of disease and anything other than “positive distant nodes”.

Patients characterized by either “positive distant nodes” and any extent of disease other than “00”, or an extent of disease in the range of 80–98 were restaged to distant. Finally, anything outside the parameters of this algorithm would be considered “in error” or “unstaged”[32]. A potential bias exists, inasmuch as men who undergo radical prostatectomies are typically “upstaged” from local to either regional or distant cancer. Thus, men who undergo this surgery are more likely to be recorded as having late-stage disease compared to those who do not. While there is no “perfect” solution to this problem, we assumed the “standard” practice of recommending radical surgery only for those with localized disease, and “backstaged” all regional or distant cancer cases to localized disease, only among men who had radical prostatectomies recorded by SEER.

We also considered plausible and measurable confounders of the potential relationship between comorbidity burden and stage at diagnosis by categorizing patients according to age at diagnosis (67–80 years old, and older than 80 years), geographic location, educational level, number of urologists per 1,000,000 residents, number of physicians per 10,000 residents, per capita income, and number of contacts with the medical care system. Contacts with the medical care system included physician visits, regardless of specialty, within the 2 years prior to cancer diagnosis. We assessed the PSA test through Common Procedure Terminology (CPT) codes in the physician-supplier file. We included PSA tests conducted within 2 years prior to the cancer diagnosis date (excluding the month of cancer diagnosis). We obtained all of the other measures from the Area Resource File[33]. High education level is the percent of persons 25 years old and older with at least 4 years of college who live in the county of the patient. Urologists are labeled “total patient care” urologists in the Health Service Area (HSA) in 1994. HSA is defined as “one or more counties that are relatively self-contained with respect to the provision of routine hospital care”[34]. Physicians are labeled “total patient care nonfederal MDs” in the HSA in 1993, 1994, 1995, corresponding to the year of cancer diagnosis. Both urologists and physicians include office-based physicians, full-time hospital staff, residents, and fellows. Per capita income is defined as per capita income (in $10,000s) in the county of residence in 1993.

We calculated unadjusted bivariate associations between stage of disease (late vs. early) and each comorbidity category and reported odds ratios (OR) and confidence intervals (CI) for these associations. Using a series of multiple logistic regression models, we also quantified the association between comorbidity burden and prostate cancer stage at diagnosis, while simultaneously controlling for the factors discussed above that might influence that relationship. We measured comorbidity burden with a set of dummy variables representing the comorbidity categories. Independent variables included age at diagnosis and the community-level variables listed above. Effect modification by race is formally tested by estimating a multiple logistic regression with race and comorbidity interactions. The backward elimination technique reduces collinearity among covariates, as insignificant comorbidity variables are eliminated, interactions first, then main effects. All covariates, other than comorbidities are retained in the model.

## RESULTS

Characteristics of the 2,489 black men and 2,587 white men studied are given in Table 1. A larger proportion of white men than black men had both localized prostate cancer, 81.7% compared to 74.8% ($p < 0.01$). For white men, the five most prevalent comorbidities were benign hypertension (46.4%), lower genitourinary disorders (42.6%), coronary artery disease (36.8%), mild-to-moderate pulmonary disease (21.5%), and dyslipidemia (21.3%). For black men, the most prevalent diseases were benign hypertension (64.5%), lower genitourinary disorders (50.9%), coronary artery disease (35.4%), diabetes (27.4%), and mild-to-moderate pulmonary disease (25.2%). Black men had higher levels of most comorbidities than white men, especially congestive heart failure (CHF) ($p < 0.01$), benign and malignant hypertension ($p < 0.01$ for both conditions), renal disease ($p < 0.01$ for both categories), and diabetes ($p < 0.01$). Black men also had a higher prevalence of multiple comorbidities, with 43.9% having five or more comorbid conditions compared to 30% for whites.

**TABLE 1**
**Characteristics of Prostate Cancer Study Sample by Race Based on SEER Program; Medicare Linked File, Patients Diagnosed 1993–1995**

| Variable | White Men n (%)[c] | Black Men n (%)[c] |
|---|---|---|
| Stage of cancer | | |
| *In situ* | 7 (0.3) | <5 (—) |
| Local[a] | 2,113 (81.7) | 1,861 (74.8) |
| Regional | 209 (8.1) | 219 (8.8) |
| Distant[a] | 258 (10.0 | 407 (16.4) |
| Number of comorbidities | | |
| None | 223 (8.6) | 214 (8.6) |
| One[a] | 369 (14.3) | 228 (9.2) |
| Two[a] | 418 (16.2) | 295 (11.9) |
| Three[a] | 461 (17.8) | 342 (13.7) |
| Four | 339 (13.1) | 318 (12.8) |
| Five or more[a] | 777 (30.0) | 1092 (43.9) |
| Coronary artery disease | 953 (36.8) | 882 (35.4) |
| Congestive heart failure[a] | 329 (12.7) | 490 (19.7) |
| Valvular disease[b] | 169 (6.5) | 124 (5.0) |
| Benign hypertension[a] | 1,199 (46.4) | 1,605 (64.5) |
| Malignant hypertension/target organ[a] | 218 (8.4) | 589 (23.7) |
| Cardiac conduction disorders[a] | 333 (12.9) | 225 (9.0) |
| Perepheral vascular disease[a] | 331 (12.8) | 432 (17.4) |
| Cerebrovascular disease | 283 (10.9) | 309 (12.4) |
| Renal disease - mild/moderate[a] | 190 (7.3) | 239 (9.6) |
| Renal disease - severe[a] | 84 (3.3) | 212 (8.5) |
| Diabetes[a] | 410 (15.9) | 683 (27.4) |
| Other endocrine[a] | 416 (16.1) | 577 (23.2) |
| Dyslipidemia[a] | 552 (21.3) | 390 (15.7) |
| Degenerative brain syndrome[b] | 72 (2.8) | 103 (4.1) |
| Psychiatric | 115 (4.5) | 132 (5.3) |
| Substance abuse[a] | 37 (1.4) | 100 (4.0) |
| Other neurologic[a] | 62 (2.4) | 137 (5.5) |
| Musculoskeletal[b] | 536 (20.7) | 574 (23.1) |
| Spine[a] | 324 (12.5) | 239 (9.6) |
| Pulmonary - mild/moderate[b] | 556 (21.5) | 626 (25.2) |
| Pulmonary - severe | 113 (4.4) | 123 (4.9) |
| Gastrointestinal - mild/moderate[a] | 266 (10.3) | 194 (7.8) |
| Gastrointestinal - severe | 87 (3.4) | 106 (4.3) |
| Lower genitourinary[a] | 1,101 (42.6) | 1,266 (50.9) |
| Hematologic nonmalignant[a] | 312 (12.1) | 563 (22.6) |
| AIDs | 0 (0.0) | <5 (—) |
| Other cancers[b] | 237 (9.2) | 282 (11.3) |

*Note:* a = Statistically significant difference between white and black men using Chi-square test ($p < 0.01$); b = statistically significant difference between white and black men using Chi-square test ($p < 0.05$); c = percentage may not add up to 100 due to rounding.

The OR (and their 95% CIs) for having cancer diagnosed at late stage (regional or distant) for each comorbidity are displayed in Table 2. An OR of less than 1.00 implies that a patient with that comorbidity was less likely to have late-stage cancer at the time of diagnosis.

**TABLE 2**
**Bivariate Associations of Comorbidities with Late-Stage[a] Prostate Cancer**

| Comorbidity | White Men (n = 2,587) | | | Black Men (n = 2,489) | | |
|---|---|---|---|---|---|---|
| | n | % Late | OR[b] (95% CI) | n | % Late | OR[b] (95% CI) |
| Coronary artery disease | 953 | 17.8 | 0.98 (0.79–1.20) | 882 | 21.8 | 0.75(0.62–0.91)[c] |
| Congestive heart failure | 329 | 22.2 | 1.35 (1.02–1.79)[d] | 490 | 27.8 | 1.18 (0.95–1.48) |
| Valvular disease | 169 | 23.1 | 1.39 (0.96–2.03) | 124 | 25.8 | 1.04 (0.69–1.57) |
| Benign hypertension | 1,199 | 16.4 | 0.81 (0.66–1.00)[d] | 1,605 | 23.3 | 0.76 (0.63–0.92)[c] |
| Malignant hypertension/target organ | 218 | 15.1 | 0.80 (0.54–1.17) | 589 | 24.5 | 0.95 (0.77–1.18) |
| Cardiac conduction disorders | 333 | 22.2 | 1.35 (1.02–1.79)[d] | 225 | 25.3 | 1015 (0.74–1.39) |
| Peripheral vascular disease | 331 | 24.5 | 1.28 (0.97–1.70) | 432 | 27.1 | 1.13 (0.89–1.43) |
| Cerebrovascular disease | 283 | 20.9 | 1.22 (0.90–1.66) | 309 | 25.2 | 1.01 (0.76–1.32) |
| Renal disease - mild/moderate | 190 | 19.0 | 1.07 (0.73–1.56) | 239 | 25.1 | 1.00 (0.73–1.36) |
| Renal disease - severe | 84 | 34.5 | 2.49 (1.57–3.94)[c] | 212 | 36.8 | 1.84 (1.37–2.47)[c] |
| Diabetes | 410 | 17.1 | 0.92 (0.70–1.22) | 683 | 22.7 | 0.83 (0.68–1.02)[d] |
| Other endocrine disorders | 416 | 17.8 | 0.98 (0.74–1.29) | 577 | 27.2 | 1.15 (0.93–1.42) |
| Dyslipidemia | 552 | 11.8 | 0.54 (0.41–0.72)[c] | 390 | 18.0 | 0.61 (0.46–0.80)[c] |
| Degenerative brain syndrome | 72 | 27.8 | 1.78 (1.05–3.01)[d] | 103 | 24.3 | 0.95 (0.60–1.51) |
| Psychiatric disorders | 115 | 19.1 | 1.08 (0.67–1.73) | 132 | 21.2 | 0.79 (0.52–1.21) |
| Substance abuse | 37 | 32.4 | 2.21 (1.10–4.43)[d] | 100 | 31.0 | 1.35 (0.88–2.09) |
| Other neurologic disorders | 62 | 29.0 | 1.89 (1.08–3.30)[d] | 137 | 27.0 | 1.11 (0.75–1.63) |
| Musculoskeletal disorders | 536 | 16.6 | 0.88 (0.68–1.14) | 574 | 25.8 | 1.04 (0.84–1.29) |
| Spine disorders | 324 | 17.3 | 0.94 (0.69–1.28) | 239 | 24.7 | 0.97 (0.71–1.33) |
| Pulmonary - mild/moderate | 556 | 19.6 | 1.14 (0.90–1.45) | 626 | 26.2 | 1.08 (0.88–1.32) |
| Pulmonary - severe | 113 | 19.5 | 1.10 (0.68–1.78) | 123 | 19.5 | 0.71 (0.45–1.12) |
| Gastrointestinal - mild/moderate | 266 | 14.7 | 0.76 (0.53–1.08) | 194 | 26.3 | 1.07 (0.76–1.49) |
| Gastrointestinal - severe | 87 | 12.6 | 0.65 (0.34–1.23) | 106 | 31.1 | 1.36 (0.90–2.08) |
| Lower genitourinary | 1,101 | 44.5 | 1.10 (0.90–1.35) | 1,266 | 25.4 | 1.02 (0.85–1.23) |
| Hematologic nonmalignant | 312 | 16.7 | 0.90 (0.65–1.23) | 563 | 26.1 | 1.07 (0.86–1.32) |
| AIDs | 0 | | | 2 | 0.00 | |
| Other cancers | 237 | 20.7 | 1.20 (0.86–1.68) | 282 | 26.6 | 1.09 (0.82-1.44) |

*Note:* a = Late-stage cancer includes regional and distant staging; b = the unadjusted OR is the odds of late-stage cancer with comorbidity of interest divided by the odds without the comorbidity of interest; c = Chi-square statistic, $p < 0.01$; d = Chi-square statistic, $p < 0.05$.

The most significant bivariate associations among white men with prostate cancer were for congestive heart failure, benign hypertension, cardiac conduction disorders, severe renal disease, dyslipidemia, degenerative brain syndrome, substance abuse, and other neurologic disorders. All except benign hypertension and dyslipidemia increased the risk of a late-stage prostate cancer diagnosis. White men with benign hypertension had only four-fifths the odds of being diagnosed with late-stage cancer compared to those without hypertension. Similarly, those with a dyslipidemia diagnosis had about half the odds of being diagnosed with late-stage cancer as those without dyslipidemia. Conversely, some comorbidities increased the odds of having prostate cancer diagnosed at a late stage, such as severe renal disease, which increased the odds by two and one-half.

Black men had a lower odds of being diagnosed with late-stage prostate cancer if they had a previous diagnosis of coronary artery disease (OR = 0.75), benign hypertension (OR = 0.76), diabetes (OR = 0.83), or dyslipidemia (OR = 0.61), but an 84% increased odds of late-stage disease with severe renal disease.

To control for the simultaneous effects of each comorbidity, as well as the effects of other sociodemographic variables already delineated, we estimated multivariate logistic regression models for white and black men combined, and for each race separately (Table 3). We used dummy variables for comorbidity, age at diagnosis, urban or rural location, per capita income, educational achievement, urologists per 1,000,000 residents, and physicians per 100,000 residents.

**TABLE 3**
**Multivariate Associations of Comorbidities with Late-Stage Prostate Cancer**

| Variable | White/Black Men (n = 5078) (OR)[b] | White Men (n = 2,587) (OR)[b] | Black Men (n = 2,489) (OR)[b] |
|---|---|---|---|
| Coronary artery disease | 0.80[d] | 0.89 | 0.73[c] |
| Congestive heart failure | 1.13 | 1.05 | 1.20 |
| Valvular disease | 1.15 | 1.40 | 0.98 |
| Benign hypertension | 0.83[d] | 0.87 | 0.83 |
| Malignant hypertension/target organ | 0.98 | 0.80 | 1.07 |
| Cardiac conduction disorders | 1.08 | 1.29 | 0.88 |
| Peripheral vascular disease | 1.24[d] | 1.24 | 1.28 |
| Cerebrovascular disease | 1.09 | 1.24 | 1.02 |
| Renal disease - mild/moderate | 0.92 | 1.03 | 0.90 |
| Renal disease - severe | 2.03[c] | 2.31[c] | 2.00[c] |
| Diabetes | 0.93 | 0.94 | 0.91 |
| Other endocrine | 1.06 | 0.96 | 1.17 |
| Dyslipidemia | 0.68[c] | 0.68[d] | 0.67[c] |
| Degenerative brain syndrome | 0.90 | 1.18 | 0.82 |
| Psychiatric | 0.78 | 0.87 | 0.69 |
| Substance abuse | 1.48 | 2.43[d] | 1.26 |
| Other neurologic | 1.12 | 1.78 | 0.96 |
| Musculoskeletal | 0.99 | 0.89 | 1.11 |
| Spine | 1.00 | 1.02 | 1.02 |
| Pulmonary - mild/moderate | 1.13 | 1.15 | 1.15 |
| Pulmonary - severe | 0.81 | 0.96 | 0.70 |
| Gastrointestinal - mild/moderate | 0.89 | 0.80 | 1.06 |
| Gastrointestinal - severe | 1.12 | 0.66 | 1.49 |
| Lower genitourinary | 1.00 | 0.93 | 1.07 |
| Hematologic nonmalignant | 0.91 | 0.72 | 1.04 |
| AIDs | <0.001 | | <0.001 |
| Other cancers | 1.17 | 1.21 | 1.13 |
| Number of comorbidities (vs. none) | | | |
| One | 0.97 | 0.95 | 0.96 |
| Two | 1.19 | 0.99 | 1.40[c] |
| Three | 0.99 | 0.78 | 1.17 |
| Four | 0.95 | 0.86 | 0.95 |
| Five or more | 1.07 | 0.88 | 1.11 |
| Race (white vs. black) | 0.68[c] | | |
| Age at diagnosis: 80 years[e] | 2.25[c] | 2.54[c] | 2.01[c] |
| Rural location[f] | 0.97 | 1.04 | 0.55 |
| Per capita income | 1.00 | 1.06 | 0.87 |
| High education[g] | 1.01 | 1.01 | 1.02 |
| Urologists/1,000,000 | 0.99[d] | 0.99 | 0.98[c] |
| Physicians/10,000 | 1.02[d] | 1.02 | 1.02 |
| Physician visits 2 years before diagnosis[h] | 1.00 | 1.00 | 0.99[d] |
| No PSA 2 years before diagnosis[i] | 0.72[c] | 0.78[d] | 0.67[c] |

*Note:* a = early-stage cancer includes *in situ* and local staging, late-stage cancer includes regional and distant staging; b = OR is the odds of late-stage cancer (probability of late-stage cancer divided by probability of early-stage cancer) with a comorbidity over the odds without; c = maximum likelihood estimate significant at $p < 0.01$; d = maximum likelihood estimate significant at $p < 0.05$; e = age 80 compared to age <80; f = rural compared to urban location; g = percentage of persons aged 25 years and older with at least 4 years of college who live in the county; h = total physician visits within 2 years of cancer diagnosis; i = PSA excludes month of diagnosis, if include month of diagnosis OR of PSA = blacks (1.22, $p < 0.05$), whites (1.01, $p < 0.90$); c statistic = 0.662 (white/black), 0.668 (whites), 0.650 (blacks).

Among men in both races, coronary artery disease, benign hypertension, and dyslipidemia were associated with a lower odds of late-stage disease at diagnosis, compared to men without these comorbidities, whereas peripheral vascular disease, severe renal disease, and substance abuse increased the odds of a late-stage prostate cancer diagnosis. Black men had a 47% increased odds of late-stage cancer compared with white men. Older men had over two times the odds of being diagnosed with late-stage cancer. A higher urologist density was associated with a lower odds of late-stage disease whereas a higher physician density was associated with an increased odds of advanced cancer

Table 3 also reports the analyses stratified by race. Black men with coronary artery disease or dyslipidemia had 72 and 67% the odds, respectively, and those with severe renal disease (primarily renal failure) had twice the odds of being diagnosed with late-stage prostate cancer than those without such diagnoses. White men with coronary artery disease, benign hypertension, or dyslipidemia had 79, 83, and 68% the odds, respectively, of being diagnosed with late-stage cancer, compared with those without these comorbidities. White men with peripheral vascular disease, severe renal disease, or substance abuse had a 24, 103, and 50% greater odds, respectively, of late-stage diagnosis compared to those without these diseases. For the most part, having multiple comorbidities is unrelated to the risk of late-stage disease, except that black men have a 40% increased odds of late-stage prostate cancer with two or more comorbidities compared to none. Both white and black men had over twice the odds of being diagnosed with late-stage disease if they were 80 years of age at the time of diagnosis compared to younger men in their race. Having a PSA test within 2 years of cancer diagnosis was associated with a lower odds of a late-stage diagnosis for all groups (0.72, 0.78, and 0.67 for all patients, whites, and blacks, respectively).

Effect modification by race is more formally analyzed in Table 4 using a multiple logistic regression, interactions among comorbidities and race, and stepwise backward elimination of nonsignificant comorbidity main effects and interactions. The results show little, if any, effect modification by race. Two comorbidities show statistically significant interactions. White men with cardiac conduction disorders (CCD) have a 40% higher odds of late-stage prostate cancers compared to men without CCD. Black men with CCD have no such increased (or decreased) risk. White men with other neurologic disorders have an 84% increased odds of late-stage cancer compared to men without these disorders. Black men with these disorders have no such increased (or decreased) risk.

## DISCUSSION

Our results suggest some significant associations between certain comorbidities and the stage at which prostate cancer is diagnosed. When both races were combined in our analyses, coronary artery disease, benign hypertension, and dyslipidemia were associated with a lower odds of late-stage cancer, compared to men without these comorbidities, whereas peripheral vascular disease, severe renal disease, and substance abuse were associated with a higher odds of late-stage cancer.

The effect of comorbid illness on late-stage prostate cancer demonstrates some limited effect modification by race. For some comorbid conditions, the effects were the same for both races. Both black and white men had two or more times the odds of late-stage disease if they had severe renal disease as a comorbidity compared to men without this disease. Likewise, dyslipidemia was associated with two-thirds the odds of late-stage disease for men of both races. On the other hand, white substance abusers had nearly two and one half times the odds of late-stage disease compared to other white men, an effect that was not significant among black men, though this effect modification was not confirmed in the race by comorbidity interaction analysis (Table 4). This same analysis showed significant increased odds of late-stage prostate cancer for white men only with either CCD or other neurologic conditions. These racial disparities are curious, as they may reflect biomedical differences in disease pathology, or, more likely, access to care, and treatment differences among races.

**TABLE 4**
**Stepwise Multivariate Logistic Models for Late-Stage[a] Prostate Cancer; 5,076 White and Black Men at Least 67 Years of Age**

| Variable | *p* Values | OR[b] | 95% CI |
|---|---|---|---|
| Main effects | | | |
| Coronary artery disease | 0.01 | 0.80 | 0.68–0.95 |
| Benign hypertension | 0.03 | 0.84 | 0.71–0.99 |
| CCD | 0.40 | 0.86 | 0.61–1.21 |
| Peripheral vascular disease | 0.03 | 1.26 | 1.03–1.55 |
| Renal disease - severe | <0.0001 | 2.06 | 1.57–2.70 |
| Dyslipidemia | 0.0001 | 0.67 | 0.54–0.82 |
| Substance abuse | 0.04 | 1.51 | 1.02–2.24 |
| Other neurologic | 0.64 | 0.91 | 0.60–1.37 |
| Gastrointestinal – severe | 0.12 | 1.43 | 0.91–1.25 |
| Interactions | | | |
| CCD*white race | | | |
| White: CCD yes vs. no | 0.03 | 1.40 | 1.03–1.89 |
| Black: CCD yes vs. no | 0.40 | 0.86 | 0.61–1.21 |
| Other neurologic | | | |
| White: other neurologic yes vs. no | 0.04 | 1.84 | 1.02–3.32 |
| Black: other neurologic yes vs. no | 0.11 | 0.91 | 0.60–1.37 |
| Gastrointestinal (GI) – severe | | | |
| White: GI – severe yes vs. no | 0.19 | 0.64 | 0.33–1.24 |
| Black: GI - severe yes vs. no | 0.12 | 1.43 | 0.91–2.25 |
| Age at diagnosis: 80 years[c] | <0.0001 | 2.24 | 1.91–2.63 |
| Rural location[d] | 0.93 | 1.01 | 0.75–1.38 |
| Per capita income | 0.85 | 0.97 | 0.74–1.28 |
| High education[e] | 0.13 | 1.01 | 1.00–1.03 |
| Urologists/1,000,000 | 0.03 | 0.99 | 0.98–1.00 |
| Physicians/10,000 | 0.02 | 1.02 | 1.00–1.04 |
| Physician visits 2 years before diagnosis[f] | 0.03 | 0.99 | 0.99–1.00 |
| PSA 2 years before diagnosis[g] | <0.0001 | 0.72 | 0.62–0.83 |
| Number of comorbidities (vs. none) | | | |
| One | 0.57 | 0.97 | 0.71–1.32 |
| Two | 0.09 | 1.19 | 0.88–1.61 |
| Three | 0.64 | 0.99 | 0.72–1.35 |
| Four | 0.38 | 0.95 | 0.67–1.32 |
| Five or more | 0.51 | 1.09 | 0.77–1.54 |

*Note:* a = early-stage cancer includes *in situ* and local staging, late-stage cancer includes regional and distant staging; b = OR is the odds of late-stage cancer (probability of late-stage cancer divided by probability of early-stage cancer) with a comorbidity over the odds without; c ≥ age. 80 compared to age <80; d = rural compared to urban location; e = percentage of persons aged 25 years and older with at least 4 years of college who live in the county; f = total physician visits within 2 years of cancer diagnosis; g = PSA excludes month of diagnosis.

At least four hypotheses have been suggested elsewhere to explain the link between comorbid illness and stage of cancer at diagnosis[35]: (1) the surveillance hypothesis — patients with coexisting disease have more frequent contact with the health care system facilitating early diagnosis, (2) the pathological hypothesis — comorbidities interact with cancer pathogenesis in such a way as to increase aggressiveness at the cellular or physiological level, (3) the competing demand hypothesis — comorbidities may distract the physician from a diagnosis of prostate cancer, and (4) the death from other causes hypothesis — patients with significant comorbidities are more likely to die from other causes and are treated less aggressively.

The surveillance hypothesis is supported by comorbidities that are associated with a decreased odds of late-stage disease, as was the case with coronary artery disease, for example. Presumably, patients with these illnesses are more likely to have regular contact with their medical providers with the regularity of contact somehow increasing the likelihood of screening.

Both pathological and competing demand hypotheses are supported by comorbidities that increase the odds of late-stage disease, such as severe renal disease or substance abuse. Whether this comorbidity actually affects the aggressiveness of cancer, whether the condition is a detraction or barrier to screening, and whether the condition lead to a poor prognosis for the patient are unclear. Severe renal disease may be associated with a compromised immune system, which could affect metastasis.

The reality is that elderly men with prostate cancer have multiple comorbidities. Among white men, 77% have a least two comorbid conditions and 30% have five or more. With black men, 82% have a least two comorbid conditions and nearly 44% have five or more. In this study with 27 comorbidities, there were 2,817 different comorbidity combinations among both races. Our study was limited in that it could not capture the subtle nuances of various patterns of comorbidities. The count of number of comorbid conditions did show significant racial differences in the burden of disease, but did not show a multiple comorbidity effect on the risk of late-stage illness, other than black men with two comorbid conditions having a 40% increased odds of late-stage disease compared to black men with no comorbidities.

This study is primarily limited by its use of Medicare claims data. Advocates of claims-based research argue that these data are useful because they are accessible, routinely collected, and represent the utilization experience of large numbers of patients. However, these data suffer from vagueness in clinical content of some ICD-9-CM codes, and the coding system may be unable to adequately account for severity of illness or the interaction of comorbidities[36]. A key limitation of the Medicare file is that coding for administrative purposes is motivated by goals inherently different from those of this type of research.

Klabunde and colleagues[37] report the “first documented attempt to develop a Charlson-like comorbidity measure using the diagnostic and procedure data contained in Medicare Part B claims” (p. 1266). We also used ambulatory claims data to supplement the data from hospital encounters. However, the prevalence of comorbidities in our research differed from that found by Klabunde, partly due to differences in comorbidity category definitions. More importantly, Klabunde’s strict algorithm omitted single physician claims for a specific comorbidity if they were not backed up by an inpatient claim, as well as claims that appeared multiple times for a specific comorbidity within a 30-day period only. Our study had no such algorithm and, as such, is more likely to overestimate than underestimate comorbidity burden. But we may still have missed less severe comorbidities that are neither reimbursed nor recorded by Medicare. Moreover, we avoided acute conditions in our 6-stage process for defining the 27 comorbidity categories, and these conditions could also have an impact on cancer diagnosis.

Our study may suffer from selection bias. We limited our study to a Medicare-eligible population of elderly men, and our results therefore may not apply to men aged less than 67 years, for whom the interaction of comorbidities and diagnosis may be entirely different. Also, the SEER program data are not generalizable to the entire U.S., as the SEER population is more urban, affluent, and has lower unemployment than the rest of the country[38]. The number (proportion) and severity of comorbidities in the SEER population may therefore not reflect those of the general U.S. population.

Our research found associations between comorbid illness and the stage at which prostate cancer is diagnosed, which are probably due to either increased surveillance through contact with the medical care system, or a pathological interaction among comorbidity and cancer. Future research in this area should be directed toward understanding why some comorbidities increase the risk of late-stage disease, which of the discussed hypotheses explains the association between late-stage disease and each comorbidity, and whether physician or patient behaviors need to be changed to increase survival and quality of life for prostate cancer patients. If some diagnoses are associated with less screening and late-stage cancer, physicians could specifically target screening for these men. If other diagnoses are associated with more screening and earlier-stage cancer, improved access to physician services might have secondary benefits for cancer prevention.

## ACKNOWLEDGMENTS

This publication/project was made possible through a cooperative agreement between the Centers for Disease Control and Prevention and the Association of Teachers of Preventive Medicine, Cooperative Agreement # U50/CCU300860 TS-319; its contents are the responsibility of the authors and do not necessarily reflect the official views of the CDC or ATPM.. The authors also appreciate the support provided by the Queensland University of Technology in Kelvin Grove, Australia where Dr. Fleming prepared this manuscript while on sabbatical. Dmitri Pavlov was a research assistant for Dr. Fleming at the University of Kentucky before his current employment. Pfizer Inc. is unassociated with this paper.
